# Cushing’s disease: adrenal steroidogenesis inhibitors

**DOI:** 10.1007/s11102-022-01262-8

**Published:** 2022-08-29

**Authors:** Rosario Pivonello, Chiara Simeoli, Nicola Di Paola, Annamaria Colao

**Affiliations:** 1 Dipartimento di Medicina Clinica e Chirurgia, Sezione di Endocrinologia, Università “Federico II” di Napoli, Naples, Italy; 2 Unesco Chair for Health Education and Sustainable Development, University “Federico II”, Naples, Italy

**Keywords:** Cushing’s disease, Metyrapone, Ketoconazole, Levoketoconazole, Osilodrostat

## Abstract

Cushing’s disease (CD), caused by an adrenocorticotropic hormone (ACTH)-secreting pituitary tumor, is the most common form of Cushing’s syndrome (CS), accounting for approximately 70% of cases. CD requires a prompt diagnosis, an adequate treatment selection, and long-term management to limit hypercortisolism duration and long-term complications and improve patient outcomes. Pituitary surgery is the first-line option, which is non-curative in one third of patients, therefore requiring additional treatments. Medical therapy has recently acquired an emerging role, with the availability of several drugs with different therapeutic targets, efficacy and safety profiles. The current review focuses on efficacy and safety of steroidogenesis inhibitors, and particularly the historical drugs, ketoconazole and metyrapone, and the novel drugs levoketoconazole and osilodrostat, which seem to offer a rapid, sustained, and effective disease control. Ketoconazole should be preferred in females and in patients without severe liver disease; levoketoconazole may offer an alternative to classical ketoconazole, appearing characterized by a higher potency and potential lower hepatotoxicity compared to ketoconazole. Metyrapone should be preferred in males and in patients without severe or uncontrolled hypokalemia. Both ketoconazole and metyrapone may be preferred for short-term more than for long-term treatment. Osilodrostat may represent the best choice for long-term treatment, in patients with poor compliance to the multiple daily administration schedule, and in patients without severe or uncontrolled hypokalemia. Steroidogenesis inhibitors may be used alone or in combination, and associated with pituitary directed drugs, to improve the efficacy of the single drugs, allowing a potential use of lower doses for each drug, and hypothetically reducing the rate of adverse events associated with the single drugs. Clinicians may tailor medical therapy on the specific clinical scenario, considering disease history together with patients’ characteristics and hypercortisolism’s degree, addressing the needs of each patient in order to improve the therapeutic outcome and to reduce the burden of illness, particularly in patients with persistent or recurrent CD.

## Introduction

**Fig. 1:**
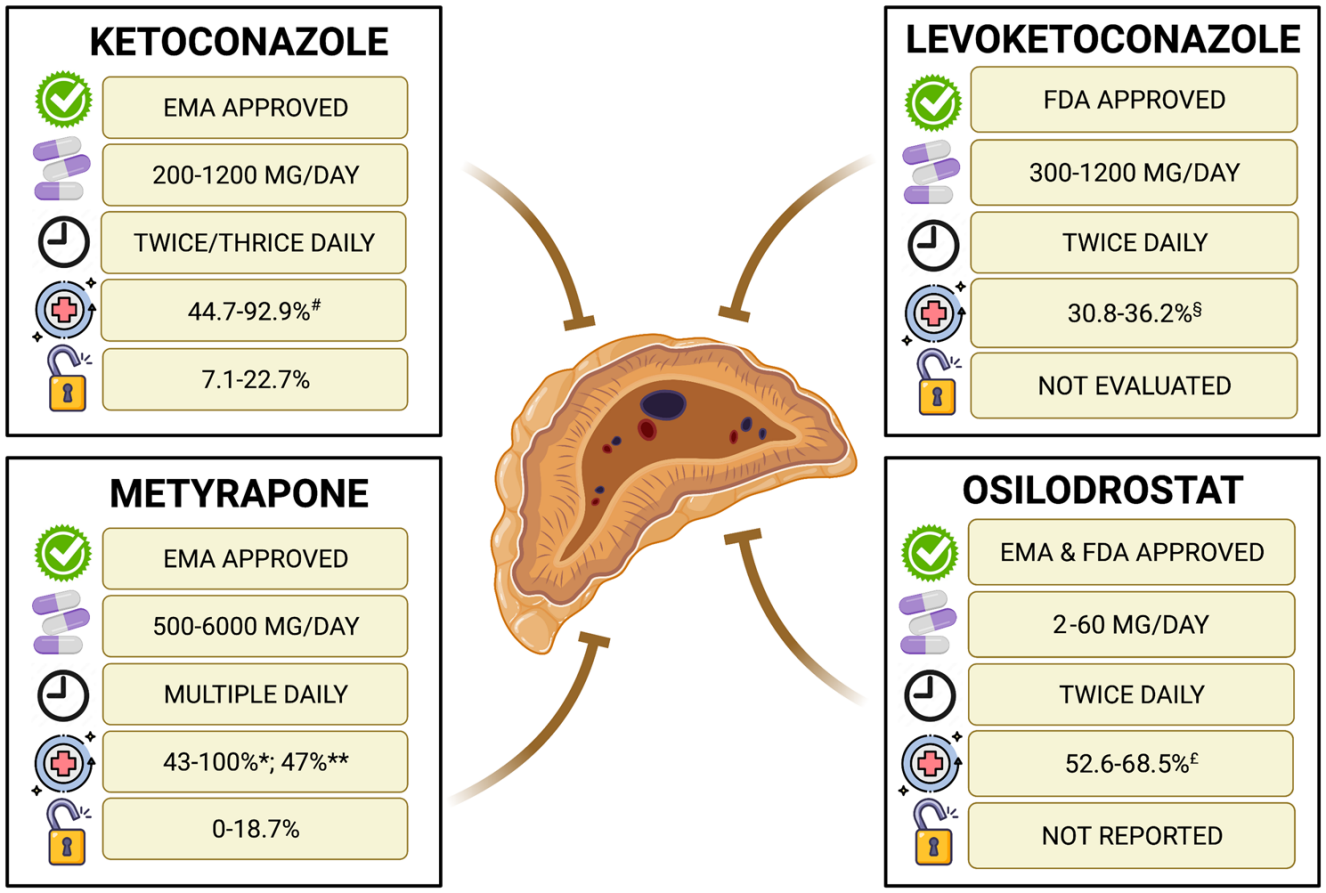
Summary of the available data regarding approval, dosage, daily administration schedule, remission rate and escape rate of the main four different steroidogenesis inhibitors. EMA: European Medicines Agency; FDA: Food and Drug Administration; # Based on data from retrospective studies [4–7]; § Based on phase III study SONICS [8] without dose up-titration and regardless of dose up-titration, respectively, at the end of the 6-month maintenance phase; * Based on data from retrospective studies [10–16]; ** Based on data from the only prospective study PROMPT [17]; £ Based on phase III study LINC 3 [21] without dose up-titration and regardless of dose up-titration, respectively, after 24 weeks of open-label treatment; ££ Based on phase III study LINC 4 [23]

Cushing’s disease (CD), caused by an adrenocorticotropic hormone (ACTH)-secreting pituitary tumor, is the most common form of Cushing’s syndrome (CS), accounting for approximately 70% of cases [1]. A prompt diagnosis and an adequate treatment are strongly necessary to limit the duration of cortisol excess exposure and the long-term complications [1]. Pituitary surgery is the first-line option in the majority of cases, but it is associated with unsuccessful outcome in an average of around one third of cases, due to immediate persistence or late recurrence of the disease [1]. Therefore, additional second-line treatments are frequently required to control the residual cortisol excess or the reappearance of the disease with a temporary or definitive solution [1]. In the CD treatment algorithm, medical therapy has recently acquired an important role due to the development of novel pharmacological compounds, potentially useful in controlling cortisol secretion [1, 2]. The current review focuses on the efficacy and safety of adrenal-directed drugs, or steroidogenesis inhibitors, particularly the drugs nowadays generally and frequently used in the clinical practice for the management of CD, due to their relevant and rapid efficacy, including the historical drugs, ketoconazole and metyrapone, and the novel drugs levoketoconazole and osilodrostat [1–3]. Table 1 provides a summary of the available data regarding the clinical efficacy and safety of the main four different steroidogenesis inhibitors. Figure 1 provides a summary of the available data regarding approval, dosage, daily administration schedule, remission rate and escape rate of the main four different steroidogenesis inhibitors.

**Table 1:** Clinical efficacy and adverse events reported in studies on the role of steroidogenesis inhibitors in the treatment of CD

	Clinical Efficacy	Adverse Events
Ketoconazole EMA approved	Clinical syndrome Hirsutism and menstrual cycles in women Body weight, blood pressure, glucose metabolism Potassium levels Muscle and bone status Psychiatric symptoms	Hepatotoxicity (10.7–18.7%) with increase in liver enzymes (2.6–18.4%); gastrointestinal disturbances (3.7–18.7%); adrenal insufficiency (5.3–18.5%); skin rash (3.6–6.2%) Gynecomastia in males (16.7%)
Levoketoconazole FDA approved	Clinical syndrome Acne, hirsutism in women Peripheral edema Body weight, glucose metabolism, lipid profile Quality of life Depressive status	Nausea (29–31.9%), headache (23–27.6%), hypokalemia (10.6–26%); hypertension (17-24%) Increase in liver enzymes (11.7–44.6%) Liver-related AEs (7.4–10.7%); QT prolongation (5.3–10.7%); hypocortisolism-related AEs (3.2–9.5%)
Metyrapone EMA approved	Clinical syndrome Body weight, blood pressure, glucose metabolism, lipid profile Muscle status Psychiatric symptoms	Hirsutism and/or acne in women (4.5–71.4%); dizziness (9.7–44.4%); nausea (5.3–33.3%); edema (6–20%) Hypertension (6–48.4%) and hypokalemia (6–13.6%)
Osilodrostat EMA & FDA approved	Body weight, blood pressure, glucose metabolism, lipid profile Quality of life Depressive status	Fatigue (28.5–58.3%); nausea (31.6–41.7%); headache (25–33.6%); diarrhea (25–31.6%); adrenal insufficiency (27.7–31.6%) Hypocortisolism-related AEs (51.1%); adrenal hormone precursors increase-related AEs (42.3%): hypokalemia (13.1%) and hypertension (12.4%); QT prolongation (3.6%); pituitary tumor enlargement (2.9%); arrhytmogenic-potentially-related episodes (0.7%)

### Ketoconazole

Ketoconazole, a racemic mixture of two enantiomers (2S,4R-ketoconazole and 2R,4S-ketoconazole) is approved from European Medicines Agency (EMA) for the treatment of CS in adults and adolescents above the age of 12 years. It is orally administered at dosages of 200–1200 mg/day, but, due to short half-life (3.3 h), it requires a twice or thrice daily administration schedule [3]. Data from prospective studies are lacking. However, data from retrospective studies, conducted in 310 CD patients, have shown that ketoconazole, at a median dosage of 620 mg/day for a median follow-up of 7.5 months, induced remission in 64.3% of patients (median 50%, range 44.7–92.9%), with 7.1–22.7% escaping after initial response [1–7]. In the largest retrospective study (FReSKO), conducted in 200 CD patients, ketoconazole at a median dosage of 600 mg/day, induced remission in 64.7% of patients treated for more than 24 months [7]. The treatment with ketoconazole is generally accompanied by an improvement in clinical syndrome and comorbidities of CS, including body weight, blood pressure, glucose metabolism, potassium levels, muscle and bone status, and psychiatric symptoms, as well as hirsutism and menstrual cycles in women [1, 2]. Data on pituitary tumor in patients treated with ketoconazole are limited. No pituitary tumor shrinkage was reported in the published literature. Conversely, in specific experiences, new tumor appeared in 13.1–13.8% of patients [6, 7]. Regarding the safety profile, the most frequently reported adverse events (AEs) were hepatotoxicity (10.7–18.7%), and particularly an increase in liver enzymes (2.6–18.4%), generally occurring early after starting treatment or at dosage increase, gastrointestinal disturbances (3.7–18.7%), adrenal insufficiency (5.3–18.5%), and skin rash (3.6–6.2%) [1]. Noteworthy, in men, gynecomastia, a potential sign of hypogonadism, was reported in 16.7% of cases in a specific study [4].

### Levoketoconazole

Levoketoconazole, the 2S,4R enantiomer of ketoconazole, is approved from Food and Drug Administration (FDA) for the treatment of CS adults for whom surgery is not an option or has not been curative. It is orally administered at dosages of 300–1200 mg/day and displays a half-life (4–6 h) longer than ketoconazole, permitting a twice daily administration schedule [2, 8]. The phase III open label study SONICS has shown that levoketoconazole, at dosages of 300–1200 mg/day, at the end of the 6-month maintenance phase, induced a complete response in 30.8% of patients without drug dose up-titration, and in 36.2% of patients regardless of dose up-titration during the maintenance phase [8]. However, considering only the 55 patients who completed the maintenance phase, levoketoconazole induced a complete response in 61.8%, and induced a partial response in 16.4%, with an overall control in 78.2%, of patients, regardless of dose up-titration [8]. The phase III double-blind, placebo-controlled, randomized withdrawal study LOGICS has shown that levoketoconazole, at dosages of 300–1200 mg/day, induced a complete response in 50% of patients at the end of randomized withdrawal, percentage significantly higher if compared to placebo (4.5%) [9]. Moreover, at the end of randomized withdrawal, significantly more patients on placebo (95.5%) achieved the primary endpoint of loss of mean urinary free cortisol (mUFC) response, defined as mUFC > 1.5 upper limit of normal range, or, for SONICS completers with mUFC above the upper limit of normal range at baseline, an increase in mUFC > 40% above the baseline value, than those who continued on levoketoconazole (40.9%) [9]. The treatment with levoketoconazole was accompanied by an improvement in clinical syndrome and comorbidities of CS, including body weight, glucose metabolism, lipid profile, peripheral edema, quality of life and depressive status, as well as hirsutism and acne in women [8, 9]. Data on pituitary tumor in patients treated with levoketoconazole are not yet available [8]. Regarding the safety profile, the most frequently reported AEs were nausea (29–31.9%), headache (23–27.6%), hypokalemia (10.6–26%), and hypertension (17–24%) [8, 9]. Noteworthy, an increase in liver enzymes was observed, and specifically alanine amino transferase (ALT) in 14.9–44.6%, gamma-glutamyl transferase (GGT) in 12.8–38.6% and aspartate amino transferase (AST) in 11.7–28.9% of cases [8, 9]. AEs were also grouped in categories of special interest, including liver-related AEs (7.4–10.7%), QT prolongation (5.3–10.7%) and hypocortisolism-related AEs (3.2–9.5%) [8, 9].

### Metyrapone

Metyrapone is approved from EMA for the treatment of CS. It is orally administered at dosages of 500–6000 mg/day, but, due to short half-life (2 h), it requires multiple daily administration schedule up to 4–6 times a day [1, 2, 3]. The great majority of published data derived from retrospective studies [10–16], with the only exception of preliminary data derived from a recent prospective study PROMPT [17]. Data from retrospective studies, conducted in a limited number of 120 CD patients, have shown that metyrapone, at a median dosage of 1750 mg/day, for a median follow-up of 5.5 months, induced remission in 71% of patients (median 75.5%, range 45.4–100%), with 0–18.7% escaping after initial response [1, 2, 10–15]. In a subsequent and largest retrospective study, conducted in 164 CS patients, metyrapone at a median dosage of 1375 mg/day in CD patients, for a median follow-up of 8 months, induced remission in 43% of patients [15]. In a subsequent and recent observational, longitudinal study, conducted in 31 CS patients, including 20 with CD, metyrapone at a median dosage of 1000 mg/day, for a follow-up of 9 months, induced remission in 70% of patients [16]. Preliminary data of the first multicenter prospective study PROMPT, conducted in 50 CS patients, showed that metyrapone, at a final median dosage of 1500 mg/day, at week 12 induced remission in 47% of patients [17]. The treatment with metyrapone was accompanied by an improvement in clinical syndrome and comorbidities of CS, including body weight, blood pressure, glucose metabolism, lipid profile, muscle status, and psychiatric symptoms [1, 2, 15–17]. Metyrapone has been sporadically used in women with CS during pregnancy, without apparent fetal complications and with hypertension and pre-eclampsia as feared maternal complications reported in anecdotical cases [3, 18]. Regarding the safety profile, the most frequently reported AEs were hirsutism and/or acne in women (4.5–71.4%), dizziness (9.7–44.4%), nausea (5.3–33.3%), and edema (6–20%) [1, 16, 17]. Noteworthy, mineralocorticoid precursors increase-related AEs, mainly represented by hypertension (6–48.4%) and hypokalemia (6–13.6%) were reported in different studies [10, 13, 14, 16, 17].

#### Osilodrostat

Osilodrostat is approved from EMA for adult CS, and from FDA for adult CD not cured by pituitary surgery or in whom pituitary surgery is not appropriate. It is orally administered at dosages of 2–60 mg/day with a higher potency and a longer half-life (4 h) than metyrapone and ketoconazole, permitting a twice daily administration schedule [3]. Different multicenter studies have evaluated the efficacy and safety of osilodrostat in patients with CD. The phase II proof-of-concept study LINC 1 has shown that osilodriostat, at dosages of 4–100 mg/day, at week 10, induced a complete response in 91.7% and partial response in 8.3%, with an overall control in 100%, of patients [19]. The phase II study LINC 2 has shown that osilodrostat, at dosages of 4–60 mg/day, at week 10, induced a complete response in 84.2% and a partial response in 5.3%, with an overall control in 89.5%, of patients, whereas, at week 22, induced a complete response in 78.9% of patients [20]. The phase III study LINC 3 has shown that osilodrostat, at dosages of 4–60 mg/day, after 24 weeks of open-label treatment, induced a complete response in 52.6% of patients without dose up-titration, and in 67.9% of patients regardless of dose up-titration [21]. At the end of the randomization withdrawal phase of 8 weeks, 86.1% of the 36 patients randomly assigned to continue osilodrostat versus 29.4% of the 34 patients randomly assigned to placebo maintained a complete response [21]. At week 48, osilodrostat induced a complete response in 66.4% of patients regardless of dose up-titration, and a partial response in 9.5% of patients, with an overall control in 75.9% of patients [21]. Noteworthy, a long-term study suggested that up to month 70 of the extension phase, osilodrostat induced a complete response in 50–88% of patients [22]. The phase III study LINC 4 has shown that osilodrostat, at dosages of 4–60 mg/day, at the end of the randomization withdrawal phase of 12 weeks, induced a complete response in 77.1% of the 48 patients randomly assigned to receive osilodrostat versus 8% of the 25 patients randomly assigned to receive placebo [23]. At week 36, osilodrostat maintained a complete response in 80.8% of patients [23]. At week 48, osilodrostat maintained a complete response in 68.5% of patients, and a partial response in 11% of patients, with an overall control in 79.5% of patients [23]. The treatment with osilodrostat was accompanied by an improvement in body weight, blood pressure, glucose metabolism, lipid profile, quality of life and depressive status [19–22]. Furthermore, in males, mean testosterone levels increased from the lower limit of normal range at baseline to the mid-normal range at week 48, with patients with hypogonadism at baseline becoming eugonadic, whereas in females, mean testosterone levels increased from the normal range at baseline to the upper limit of normal range at week 48 [21]; however, long-term data seem to suggest that in females mean testosterone levels increased from baseline to week 22, reaching as average the normal at the last assessment [22]. Considering patients with measurable pituitary tumor both at baseline and at follow-up visit, an increase or a decrease in tumor volume ≥ 20% was observed in 30.3–37.5% and in 28.8–32.8% of patients, respectively, after 24–48 weeks, during LINC 3 study [21]. Regarding the safety profile, the most frequently reported AEs were fatigue (28.5–58.3%), nausea (31.6–41.7%), headache (25–33.6%), diarrhea (25–31.6%) and adrenal insufficiency (27.7–31.6%) [19–21]. AEs were also grouped in categories of special interest, including hypocortisolism-related AEs (51.1%), adrenal hormone precursors increase-related AEs (42.3%), mainly represented by hypokalemia (13.1%) and hypertension (12.4%), together with QT prolongation (3.6%), pituitary tumor enlargement (2.9%), and arrhytmogenic-potentially-related episodes (0.7%) [21].

#### Combined treatment

Although there are few rigorous data available in literature supporting specific regimens of combined treatment, a combination of steroidogenesis inhibitors may be used to improve the efficacy of single drugs, because of the additional or synergic actions, putatively allowing a potential use of lower doses for each drug, and therefore hypothetically reducing the rate of AEs, associated with the single drugs. Particularly, combination of ketoconazole at dosages of 400–1200 mg/day, metyrapone at dosages of 3–4.5 g/day, and mitotane at dosages of 3–5 g/day induced remission in 63.6% of CS patients [24]. The treatment was accompanied by an improvement in clinical syndrome and comorbidities, including body weight, blood pressure and glucose metabolism [24]. Regarding the safety profile, the most frequently reported AEs were hypokalemia (100%), mainly initially experienced as episodes, increase in liver enzymes, and specifically AST, ALT and GGT (18.2–81.8%), nausea and vomiting (63.6%) and adrenal insufficiency (36.4%) [24]. Moreover, considering that in CD targeting the pituitary tumor is a key treatment goal, a combination of steroidogenesis inhibitors, such as ketoconazole, with pituitary directed drugs, such as the somatostatin analogue pasireotide, officially approved, and the dopamine agonist cabergoline, used off-label, may be used to concomitantly achieve the fast resolution of hypercortisolism, acting at different levels with different mechanisms of action, and tumor growth control [25–27]. Combination of cabergoline at dosages of 0.5–3 mg/week and ketoconazole at dosages of 200–600 mg/day induced remission in 66.7–78.6% of CD patients [25, 26]. The treatment was accompanied by an improvement in body weight, waist circumference, blood pressure and glucose metabolism [25], with a mild increase in liver enzymes (11.1%) as the only reported AE [26]. Combination of pasireotide at dosages of 300–750 μg/day, cabergoline at dosages of 2–6 mg/week, and ketoconazole at a dosage of 600 mg/day induced remission in 88.2% of CD patients, using a stepwise approach with pasireotide as the starting treatment and cabergoline and ketoconazole as first and second additional treatment, respectively [27]. The treatment was accompanied by an improvement in clinical syndrome, including body weight, waist circumference and blood pressure [27]. Regarding the safety profile, the only reported AEs were disturbance of glucose metabolism and serum insulin-like growth factor 1 (IGF1) levels decrease below the normal range (52.9%) [27].

## Opinion

Nowadays several drugs with different targets and mechanisms of action can be used to treat CS, and no absolute recommendations are available to guide the choice. However, some suggestions may be provided, based on available data and personal experience. Steroidogenesis inhibitors are generally effective treatments, and due to their rapid action, represent a prompt solution for CS, especially in case of severe disease, although they do not directly target the pituitary tumor, therefore representing a palliative option in CD, where the monitoring of the pituitary tumor is required together with the confirmation of a stable control of cortisol secretion during the treatment course [1, 2]. Ketoconazole should be preferred in females, due to the possible impact on testis function, or at least in males with clearly normal androgen production, maintaining a close monitoring of androgen levels and function, as well as in patients without severe liver disease, due to the potential hepatotoxicity, maintaining a close monitoring of liver enzymes. Based on recent available data, levoketoconazole may offer an alternative to classical ketoconazole, being preferred in the same categories of patients, although preclinical studies suggest it to be characterized by a higher potency and potential lower hepatotoxicity compared to ketoconazole. On the other hand, metyrapone should be preferred in males, due to the potential induction or worsening of clinical hyperandrogenism in females, or at least in females in whom clinical hyperandrogenism does not appear as an issue, maintaining a close monitoring of androgen levels and clinical hyperandrogenism signs, as well as in patients without severe or uncontrolled hypokalemia, due to the possible induction of lowering potassium levels, which need to be monitored during the treatment course. The multiple daily administration schedule associated with treatment with both ketoconazole and metyrapone may represent a limitation in patients with poor compliance. Moreover, they are generally preferred for short-term more than for long-term treatment, or in patients available to perform frequent routine assessments, due to the possible occurrence of treatment escape. Osilodrostat may represent the best choice for long-term treatment due to its long-term efficacy, at low-medium and stable doses in the majority of cases, without apparent evidence of escape. Moreover, due to its twice-daily oral administration and its good safety profile, osilodrostat may be comfortable for patients, potentially improving treatment compliance, with a potential positive impact on success rate. However, osilodrostat should be preferred in patients without severe or uncontrolled hypokalemia, due to the possible induction of lowering potassium levels, which need to be monitored during the treatment course. Furthermore, due to the occurrence of cortisol withdrawal syndrome, or adrenal insufficiency, probably due to the great potency of osilodrostat, the treatment should be associated with an early and frequent clinical monitoring, especially in patients with mild disease and particularly in the initial weeks of treatment.

In clinical practice, clinicians should select a specific drug addressing the needs of each patient in a “tailored” approach, in order to improve the therapeutic outcome and to reduce the burden of illness. A direct comparison between steroidogenesis inhibitors is difficult due to the absence of head-to-head trials, and the presence of studies which are different in terms of design, inclusion and exclusion criteria, and primary endpoints [2]. However, rapidity of action and remission rates, in terms of cortisol normalization, may be considered two key factors in determining the potency of the different drugs. Based on available data, metyrapone and osilodrostat appear to have the fastest action, with a typical response within hours, while ketoconazole, and consequently levoketoconazole, within days [2]. Moreover, based on remission rates, osilodrostat appears to have the highest efficacy, followed by metyrapone, ketoconazole and levoketoconazole. A direct comparison between the racemic ketoconazole and the selective levoketoconazole is not available at clinical levels; however, experimental studies seem to suggest that levoketoconazole displays a higher potency in inhibiting adrenal enzymes compared to dextroketoconazole and to racemic ketoconazole, potentially allowing lower doses of levoketoconazole to achieve the same efficacy of ketoconazole [28, 29]. Moreover, levoketoconazole showed a lower potency toward liver enzymes inhibition compared to dextroketoconazole, suggesting an improved safety profile of levoketoconazole on liver function [28].

Treatment response to steroidogenesis inhibitors may be monitored based on a combination of clinical endpoints, mainly in terms of changes in signs and symptoms, body weight, blood pressure, glucose metabolism, and quality of life, as well as biochemical endpoints, where both UFC and late-night salivary cortisol appear to be the preferred biomarkers to monitor the treatment response, the first one offering, with limitations related to the variability, information on the daily cortisol production, whereas the second one, with some limitations related to collection conditions, information on the restoration of cortisol rhythm [2]. Specifically, for steroidogenesis inhibitors associated with adrenal hormone precursors increase, such as metyrapone and osilodrostat, mass spectrometry should be the ideal technique to assess cortisol levels, to reduce the cross-reactivity with adrenal hormone precursors. Conversely, morning serum cortisol may be useful in patients taking relevant doses of drugs, especially in the evening, and appears the preferred biomarker to monitor the eventual appearance of adrenal insufficiency [2].

Guidelines suggest changing treatment if cortisol concentrations are persistently elevated after 2–3 months on maximum tolerated doses [2]. If the treatment response is only partially achieved, with cortisol reduction without normalization, or in case of severe disease, a combination therapy approach may be considered. Considering the potential pituitary tumor growth concern, a careful monitoring of ACTH levels and a pituitary magnetic resonance imaging every 6–12 months after initiating treatment, and thereafter every few years, are suggested [2].

All steroidogenesis inhibitors may be associated to the risk of developing adrenal insufficiency due to transient overtreatment, especially with potent drugs and particularly during dose-titration period performed to achieve cortisol normalization, although a clinical condition suggestive of adrenal insufficiency may be induced by a rapid decrease of cortisol levels, often induced by the most potent and rapid drugs, representing however a cortisol withdrawal syndrome more than a real adrenal insufficiency. In patients with severe disease, requiring starting treatment with high doses, and in patients not eligible for surgery, a block-and-replace regimen, consisting in treating patients with a combined medical approach with adrenal steroidogenesis inhibitors and exogenous glucocorticoids, may be considered. This approach, particularly useful in case of infrequent routine assessments, may reduce the risk of developing adrenal insufficiency, although caution is required to avoid iatrogenic CS [2].

In conclusion, the landscape of medical therapy in CS has been recently enriched by several drugs with different therapeutic targets, efficacy and safety profiles. Therefore, clinicians may individualize medical therapy based on the specific clinical scenario, including disease history, patients’ characteristics and hypercortisolism’s degree, addressing the needs of each patient in a more tailored approach, in order to improve the therapeutic outcome and to reduce the burden of illness, particularly in patients with persistent or recurrent CD.

## Data Availability

Not applicable.
